# Retinal circulation time/arm-to-retina time ratio in the fluorescein angiography to evaluate retina-specific hemodynamics

**DOI:** 10.1038/s41598-022-21117-3

**Published:** 2022-10-19

**Authors:** Yasuaki Mushiga, Norihiro Nagai, Yoko Ozawa

**Affiliations:** 1 Department of Ophthalmology, St. Luke’s International Hospital, 9-1 Akashi-cho, Chuo-ku, Tokyo, 104-8560 Japan; 2 St. Luke’s International University, 9-1 Akashi-cho, Chuo-ku, Tokyo, 104-8560 Japan; 3 Laboratory of Retinal Cell Biology, Department of Ophthalmology, Keio University School of Medicine, 35 Shinanomachi, Shinjuku-ku, Tokyo, 160-8582 Japan; 4 Department of Ophthalmology, Keio University School of Medicine, 35 Shinanomachi, Shinjuku-ku, Tokyo, 160-8582 Japan

**Keywords:** Imaging, Eye diseases

## Abstract

To evaluate dynamic circulatory flow in the retinal or choroidal circulatory disease, we retrospectively reviewed medical charts of 128 eyes of 128 patients who underwent video recorded fluorescein angiography (FA), at Department of Ophthalmology, St Luke’s International Hospital, between April and September 2020. Mean age was 64.2 ± 14.0 (range 37–93) years, and 87 (67.9%) patients were men. Mean arm-to-retina (AR) time was 16.2 ± 4.1 s, and mean retinal circulation (RC) time was 10.9 ± 3.3 s. Mean RC time/AR time (RC/AR) ratio was 0.69 ± 0.22. AR time was correlated with age, whereas RC time was not. RC time was positively correlated with AR time (R = 0.360, P = 0.017). Moreover, mean RC time was significantly longer, and RC/AR ratio was greater, in the retinal-disease group after adjusting for age and sex. Patients who had an RC/AR ratio ≥ 0.8 more frequently presented with retinal diseases. RC time and RC/AR ratio were negatively correlated with systolic blood pressure only in the retinal-disease group. Given that AR time reflects systemic hemodynamics, RC time, which reflects local circulatory fluency, was influenced by the systemic circulatory condition. Moreover, RC/AR ratio revealed that circulatory changes peculiar to the retina may also be involved in retinal-disease pathogenesis. This study may help elucidate the mechanisms of retinal diseases and assist in diagnosis, although further studies are required.

## Introduction

Changes in vascular circulatory flow in the retina and choroid are observed in various vision-threatening diseases. Examining changes in the circulatory fluency would be of value to evaluate the pathogenesis of the vascular and circulatory lesions.

Fluorescein angiography (FA) has long been used to diagnose and evaluate ocular vascular diseases^1,2^. Optical coherence tomography angiography, a recently developed modality, is routinely employed in the daily clinic because of its low invasiveness. In optical coherence tomography angiography, the vascular flow path can be traced to draw vascular images. In contrast, the dynamic circulatory flow speed can be visualized with FA recorded using video systems.

FA is clinically performed to diagnose retinal circulatory diseases, such as diabetic retinopathy (DR), and retinal vein occlusion (RVO). DR has been estimated to affect 4 million people worldwide^3^, and is a microangiopathy characterized by dysregulated vascular regeneration following pericyte loss and inflammation^4^. RVO was observed in 2% of participants older than 40 years in a Japanese cohort^5^. Although systemic factors, such as hypertension and high hematocrit, mainly increase the risk for RVO, local conditions, such as glaucoma, may also be culpable^6^. FA is also performed in choroidal circulatory diseases, such as age-related macular degeneration (AMD), central serous chorioretinopathy (CSC), and myopic choroidal neovascularization (mCNV). AMD affects > 1% of individuals older than 50 years worldwide^5,7,8^. Of the two AMD types, wet AMD is characterized by macular neovascularization mostly originating from the choroidal vasculature. CSC is a common eye disease characterized by serous retinal detachment and categorized in pachychoroid disease as well as some subtypes of wet AMD^9^. mCNV is another blinding disease and has received attention because of the worldwide increase in myopia as reported in a 2015 News and Comments article in *Nature* titled “The Myopia Boom”.

Information obtained from FA images includes data on non-perfusion areas and/or vascular obstruction (ischemic changes), vascular hyperpermeability (leakage), and retinal neovascularization. In addition, we can evaluate circulatory fluency if the images are obtained using video recordings. We can measure arm-to-retina time (AR time), which reflects the time from the administration of fluorescein into the antecubital vein to the time the fluorescein reaches the retinal arteries, and retinal circulation time (RC time), which reflects the time from the dye first appearing in the retina to the time the dye returns to the vein at the optic disc. AR time is influenced by the hemodynamics of systemic macrocirculation, and RC time reflects local retinal microcirculation^10^.

In the current study, we compared the AR and RC times in retinal and choroidal circulatory diseases to deepen the understanding of the relationships between systemic and local retinal circulation and of the impact of local retinal changes in retinal circulatory diseases. This study will help elucidate the pathogenesis of retinal and choroidal circulatory diseases and assist in diagnosing whether patients have local retinal changes in addition to influences of systemic hemodynamics.

## Results

### Characteristics of the patients

Data of 128 eyes of 128 patients were included in the analyses. The mean age of the patients who underwent FA was 64.2 ± 14.0 (range 37 to 93) years (Table 1). Eighty-seven (67.9%) participants were men. The mean best-corrected visual acuity (BCVA) was 0.19 ± 0.38 (range − 0.08 to 2.00), and the mean intraocular pressure was 14.0 ± 3.1 (9 to 25) mmHg. The mean AR time was 16.2 ± 4.1 (7.96 to 25.92) s, and the mean RC time was 10.9 ± 3.3 (5.9 to 28.8) s. The mean RC/AR ratio was 0.69 ± 0.22 (0.28 to 1.43). Mean AR time was longer in men (17.2 ± 4.0 s) than women (14.1 ± 3.5 s, P = 0.002), and mean RC time was longer in men (11.4 ± 3.5 s) than women (9.6 ± 2.6 s, P < 0.001), while there was no differences in RC/AR time according to sex (P = 0.243). Sixty-three (49.3%) patients had retinal diseases, and 65 (50.7%) patients had choroidal diseases. Retinal diseases included DR (37 eyes, 28.9%) and branch (22 eyes, 17.1%) and central (4 eyes, 3.1%) RVO, and choroidal diseases included AMD (37 eyes, 28.9%), CSC (18 eyes, 14.0%), and mCNV (10 eyes, 7.8%). Data in each disease were shown in Supplementary Table 1. Note that the retinal veins were recirculated on FA in all patients with RVO, and there were no particular differences in the RC times per the four branches of the retinal veins in each individual except for one patient with branch RVO; the respective RC time of affected and unaffected area in the one exceptional patient was 17.0 and 9.2 s, and the difference was 7.8 s, however, the mean difference of the affected and unaffected area in the other patients with branch RVO were 0.08 ± 0.24 s. The AR time was positively correlated with age (R = 0.174, P = 0.049); however, RC time was not (R = 0.009, P = 0.922) (Supplementary Fig. 1). RC/AR ratio was also not correlated with age (R = − 0.138, P = 0.120). HbA1c was recorded only 20 out of 37 patients with DR in the current study, and there was no trend of correlation between HbA1c and the AR time (P = 0.773 R = − 0.069), and the RC time (P = 0.888, R = − 0.034) at least in the 20 patients.

**Table 1:** Characteristics of the patients.

Characteristic	All patients
Number of patients	128
Age (years old)	64.2 ± 14.0 (37 to 93)
Sex (men [%])	87 (68)
Best-corrected visual acuity (LogMAR)	0.19 ± 0.38 (− 0.08 to 2.00)
Intraocular pressure (mmHg)	14.0 ± 3.1 (9.0 to 25.0)
Arm-to-retina (AR) time (s)	16.2 ± 4.1 (7.96 to 25.92)
Retinal circulation (RC) time (s)	10.9 ± 3.3 (5.9 to 28.8)
Retinal diseases (eyes [%])	63 (49)
RC/AR ratio	0.69 ± 0.22 (0.28 to 1.43)
Systolic blood pressure (mmHg)	137.9 ± 19.0 (97 to 198)
Diastolic blood pressure (mmHg)	76.2 ± 12.4 (51 to 118)

Data are shown in mean ± standard error (range).

*AR time* arm-to-retina time, *RC time* retinal circulation time, *RC/AR ratio* RC time/AR time ratio.

### Differences in the AR and RC times in the retinal and choroidal diseases

**Figure 1:**
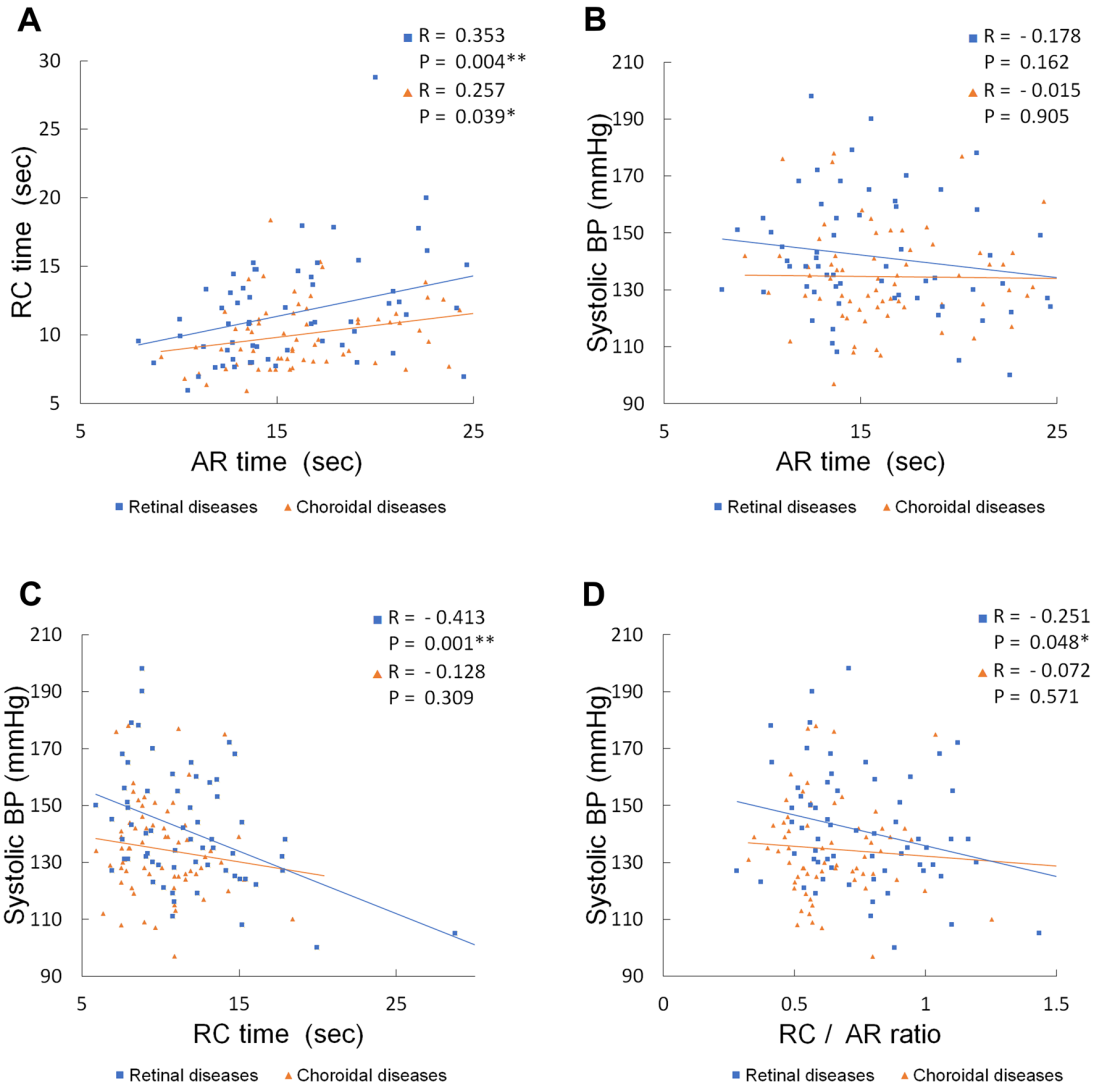
Correlation between arm-to-retina (AR) time and retinal circulation (RC) time, and systolic blood pressure (BP) and each parameter in patients with retinal diseases and choroidal diseases. (**A**) A positive correlation was observed between AR time and RC time in both groups. (**B**–**D**) Correlations between systolic blood pressure (BP) and each parameter. Although there was no correlation between systolic BP and AR time in either retinal- or choroidal-disease group (**B**), a negative correlation was observed between systolic BP and RC time (**C**), and systolic BP and RC/AR ratio (**D**) only in the retinal-disease group. **P < 0.01, *P < 0.05.

RC time was positively correlated with AR time in all patients (R = 0.360, P = 0.017) (Fig. 1). A significant correlation was also observed when the patients were divided into a retinal-disease group (R = 0.353, P = 0.004) and a choroidal-disease group (R = 0.257, P = 0.039) (Fig. 1A).

However, RC time was significantly more prolonged in patients with retinal diseases than in those with choroidal diseases, whereas AR time did not differ between the groups (Table 2). The RC/AR ratio was significantly greater in the retinal-disease group. There were no significant differences in age, sex, BCVA, and IOP between the groups (Table 2).

**Table 2:** Differences in the patients with retinal diseases and choroidal diseases.

	Retinal diseases	Choroidal diseases	P value
Number of patients	63	65	–
Age (years old)	62.7 ± 12.1 (40 to 89)	65.7 ± 15.5 (37 to 93)	0.246
Sex (men [%])	43 (66)	44 (70)	0.946
BCVA (LogMAR)	0.18 ± 0.34 (− 0.08 to 1.70)	0.19 ± 0.42 (− 0.08 to 2.00)	0.894
Intraocular pressure (mmHg)	13.9 ± 3.4 (9.0 to 25.0)	14.0 ± 2.9 (9.0 to 21.0)	0.601
AR time (s)	16.2 ± 4.6 (8.0 to 25.9)	16.3 ± 3.6 (9.1 to 24.3)	0.534
RC time (s)	11.7 ± 3.8 (5.9 to 28.8)	10.0 ± 2.4 (5.9 to 18.4)	0.008**
RC/AR ratio	0.75 ± 0.23	0.64 ± 0.18	0.002**
Systolic blood pressure (mmHg)	141.3 ± 20.4 (100 to 198)	134.7 ± 17.1 (97 to 178)	0.072
Diastolic blood pressure (mmHg)	77.2 ± 12.1 (57 to 108)	75.2 ± 12.7 (51 to 118)	0.294

Data are shown in mean ± standard error (range). *AR time* arm-to-retina time, *RC time* retinal circulation time, *RC/AR ratio* RC time/AR time ratio. **P < 0.01.

Although there were no correlation between systolic blood pressure and AR time in either group (retinal-disease group, R = − 0.178, P = 0.162; choroidal-disease group, R = − 0.015, P = 0.905) (Fig. 1B), there was a negative correlation between systolic blood pressure, and RC time (R = − 0.413, P = 0.001) (Fig. 1C) and RC/AR ratio (R = − 0.251, P = 0.048) (Fig. 1D) in the retinal-disease group, while not in the choroidal-disease group (RC time, R = − 0.128, P = 0.309; RC/AR time, R = − 0.072, P = 0.572).

### Circulatory flow differences between retinal and choroidal diseases

Next, the factors reflecting differences between retinal and choroidal diseases were analyzed using logistic regression analyses. Crude analysis showed that RC time (P = 0.006) and the RC/AR ratio (P = 0.003) significantly differed between the groups (Table 3). After adjusting for age and sex, RC time (odds ratio, 0.820; 95% confidence interval, 0.716–0.939; P = 0.004) and the RC/AR ratio (odds ratio, 0.680; 95% confidence interval, 0.011–0.431; P = 0.004) remained significantly different between the groups (Table 3).

**Table 3:** Factors which reflect the differences of retinal diseases and choroidal diseases.

	Crude			Multivariate, adjusted		
	OR	95% CI	P value	OR	95% CI	P value
Age	1.016	0.990–1.042	0.226	–	–	–
Sex	0.975	0.464–2.048	0.946	–	–	–
BCVA (LogMAR)	1.100	0.041–2.742	0.839	1.003	0.474–2.123	0.993
Intraocular pressure	1.015	0.908–1.134	0.798	1.019	0.992–1.047	0.173
AR time (s)	1.005	0.923–1.094	0.912	0.994	0.905–1.092	0.906
RC time (s)	0.834	0.733–0.949	0.006**	0.82	0.716–0.939	0.004**
RC/AR ratio	0.063	0.010–0.391	0.003**	0.68	0.011–0.431	0.004**

Logistic regression analyses. In the multivariable analysis, potential factors were adjusted for age and sex. *AR time* arm-to-retina time, *RC time* retinal circulation time, *RC/AR ratio* RC time/AR time ratio, *OR* odds ratio, *CI* confidence interval. *P < 0.05, **P < 0.01.

### RC/AR ratio of the retinal and choroidal diseases

**Figure 2:**
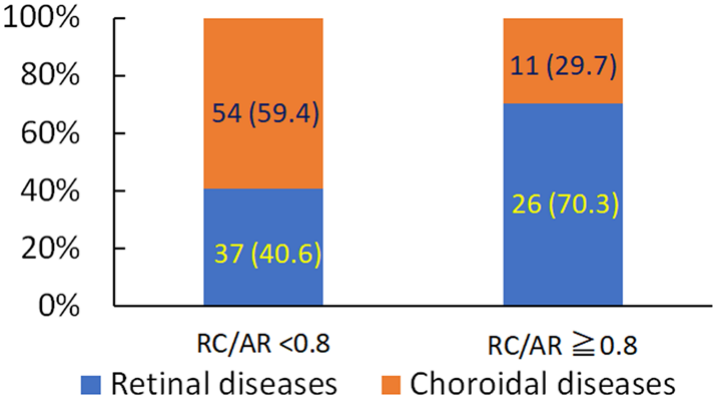
Numbers of patients with retinal and choroidal diseases who had a retinal circulation time/arm-to-retina time ratio < 0.8 or ≥ 0.8. Eyes (%). *RC/AR* retinal circulation time/arm-to-retina time ratio.

We further analyzed the RC/AR ratio across individual patients. The Youden index of the ROC curve (AUC value, 0.657) was 0.79 (Supplementary Fig. 2), and we analyzed the patients with or without RC/AR ratio ≥ 0.8. We found that patients who had an RC/AR ratio ≥ 0.8 more frequently presented with retinal diseases than with choroidal diseases (Fig. 2).

## Discussion

In this study, we examined dynamic circulatory flow with video recorded FA in retinal and choroidal circulatory diseases. The AR time was correlated with age, whereas RC time was not. There were positive correlations between the AR and RC times in both retinal and choroidal vascular diseases. The RC time and RC/AR ratio were negatively correlated with systolic blood pressure only in the retinal-disease group. The mean AR time was comparable between the retinal- and choroidal-disease groups. However, the mean RC time was significantly longer, and the RC/AR ratio was greater, in the retinal-disease group after adjusting for age and sex. Patients with an RC/AR ratio ≥ 0.8 more frequently presented with retinal diseases.

The mean AR time was approximately 16 s in the overall sample, and it was also around 16 s in both retinal and choroidal disease groups; the values were close to the upper limit of the normal range, which is reportedly 10–15 s^11^. The correlation between AR time and age would be at least in part related to atherosclerosis^12^, given that AR time reflects the systemic hemodynamic condition. Atherosclerosis, which progresses with age, is assumed to result from macrophage activation and mitochondrial deficiency in the vascular endothelial cells^12^ and can be accelerated by high serum cholesterol levels and hypertension^13–15^. This correlation was observed in both the retinal- and choroidal-disease groups, suggesting that systemic vascular conditions, such as age-related systemic atherosclerosis, in patients with either disease may be similar. In fact, hypertension is a known risk factor of most diseases included in the current study^16–20^.

In contrast, RC time was not correlated with age in the current study. Fundus photographs have been used to detect the risk of systemic hypertension and arteriosclerosis in several settings, such as during health checkups. Ultra-wide-field pseudo-color images were recently reported to be valuable for predicting vascular aging, which is generally evaluated based on brachial–ankle pulse-wave velocity, using deep learning^21^. Thus, retinal vascular appearance could represent one aspect of the systemic vascular condition. However, RC time, which reflects circulatory function and not just outward appearance, may be affected by factors other than systemic conditions, such as local vascular structures and/or differences related to the nature of the structures, e.g., arteries/veins or arterioles/venules^22^, and/or other second hit stimuli peculiar to the retina. The retina receives light stimuli, and whether light exposure, which can induce retinal inflammation^23,24^, could be involved in the second hit stimuli would be a topic for future research.

More interestingly, RC time was longer in the retinal-disease group. Because sex can also affect vascular aging^25^, we adjusted for both sex and age to assess the impact of RC time in retinal diseases and determine whether there was a significant difference in RC time between the retinal- and choroidal-disease groups. Sex differences are most frequently reported in ocular diseases associated with impaired ocular blood flow, such as AMD and DR, most likely because of hormonal differences^26^.

Previous reports has shown that patients with diabetes whose hemoglobin A1c was greater than 9.5 g/dl showed longer RC time than those whose HbA1c was lower than 8.0 g/dl, and related to the blood sugar levels, whereas AR time was comparable between the groups^27^. Diabetes causes pericyte loss and resulting micro-vascular damage^28^, which induces recruitment of inflammatory cells^29^ to finally cause circulatory insufficiency. HbA1c in the patients with DR was not fully assessed in the current study, and this point would be further studied in the future. In branch RVO, although it has been reported, using laser speckle flowgraphy, that there was increase in resistivity in the retinal artery of the affected area^30^, we did not observe differences in the RC time of the affected and unaffected areas, except for one eye; suggesting the possibility that the vascular flow of the unaffected area would be also influenced, i.e., by hypoxia-related inflammatory cytokines and/or neurogenic controls, which may have reduced the fluency of the entire retinal circulation. This point would be further assessed in the future. In central RVO, it has been reported that RC time is prolonged while AR time is not, and RC time longer than 20 s increased the risk of neovascularization on the iris^31^. RC time could be a marker of pathological retinal conditions most likely related to relative hypoxia, although further studies are required.

The difference in the meaning of the AR and RC times in the pathogenesis was more obvious when we calculated the RC/AR ratio. Although RC time was correlated with AR time, comparison of the RC and AR times in each individual revealed retina-specific hemodynamic changes, i.e., evaluation of the RC/AR ratio emphasized the independent impact of retinal local hemodynamic changes apart from systemic hemodynamic changes. The RC/AR ratio was significantly greater in the retinal-disease group after adjusting for age and sex. Moreover, an RC/AR ratio ≥ 0.8 was more frequently observed in patients with retinal diseases. The AR and RC times were correlated across individual patients. However, the differences in the RC/AR ratio supported the notion that second hit stimuli may be implicated in the development of retinal vascular diseases in addition to the influence of pathological systemic hemodynamic conditions, such as hypertension. In contrast, retinal circulation would be less influenced in choroidal diseases.

In fact, the RC time and RC/AR ratio were negatively correlated with systolic blood pressure only in the retinal-disease group. The normal retinal hemodynamic response to increased perfusion pressure is an increase in vascular resistance intrinsically present in smooth muscle cells^32^. If the blood pressure is elevated, vascular resistance would be elevated, which results in delayed perfusion and longer RC time. However, the current results were opposite. The autoregulation system of the retinal vessels might have been disordered in the retinal-disease group, and this could be one of the peculiar changes described above, although further studies are required.

Taken together, the pathogenesis of retinal vascular diseases may implicate both systemic circulatory, i.e., macroangiopathy, and retina-specific circulatory i.e., microangiopathy, etiologies^33^. The factors originated in the retina which promote retinal blood-flow disorders would be a future research interest. Measuring the AR and RC times, and the RC/AR ratio may help elucidate the pathogeneses and/or diagnose the retinal and choroidal diseases, although further studies are required.

Limitations of the current study included the relatively small sample size, retrospective analyses, involvement of several diseases in the retinal- and choroidal-disease groups, and absence of data from healthy participants. However, FA is a relatively invasive examination, and we may not be able to obtain data from individuals who have no medical necessity to undergo FA. The video recording was limited to the first 40 s, and when the AR time was long, the RC was determined via the subsequently acquired sequential photographs; in such cases, RC time was determined just before the dye intensity of the four major veins became indistinguishable, and may have been underestimated. However, this was only observed in patients with diabetes; nonetheless, the retinal-disease group showed longer RC times. The needle of the dye injection was 22 or 24 gages according to the condition of the vein at the injection insertion point in individuals; this may have affected RC and AR times, however, may not have affected RC/AR time ratio.

In summary, patients with retinal diseases had longer RC time and a greater RC/AR ratio than those with choroidal diseases. Given that RC time, which reflects local circulatory fluency, was correlated to AR time, which reflects systemic hemodynamics, local retinal circulatory fluency was, at least in part, influenced by systemic circulatory conditions. Moreover, the greater RC/AR ratio in retinal vascular diseases suggested that circulatory changes peculiar to the retina may also be involved in the pathogenesis of retinal diseases. Measuring the RC/AR ratio may allow evaluation of retina-specific hemodynamic changes, and help elucidate the mechanisms of retinal diseases and assist in diagnosis, although further studies are required.

## Methods

The study adhered to the tenets of the Declaration of Helsinki, was approved by the Ethics Committee of the St Luke’s International University (Tokyo, Japan; 20-R048), and was registered in University hospital Medical Information Network (Tokyo, Japan; UMIN000040310). Informed consent was obtained from all participants.

### Participants

Medical charts of patients who underwent FA (Heidelberg Engineering SPECTRALIS^®^) at the Department of Ophthalmology, St Luke’s International Hospital, between April 2020 and September 2020, and were diagnosed with retinal or choroidal vascular diseases were retrospectively reviewed. We excluded patients who had other than the above-described diseases, such as uveitis, and those with diseases of unknown etiology.

### Ocular examinations

All patients underwent BCVA measurements using refraction testing, slit-lamp examination, and binocular indirect ophthalmoscopy after pupil dilation with 0.5% tropicamide.

### AR time and RC time measurements using FA

FA was performed using Heidelberg Engineering SPECTRALIS^®^. The dye, fluorescite (3 ml; Novartis Pharma K.K., Tokyo, Japan) was injected using 22 or 24 gages of the needle. AR time was measured from the time the dye entered the injection insertion point to the time the dye reached the retinal artery^34^. RC time was measured from the time the dye reached the retinal artery to the time the dye reached the retinal vein at the optic disc, and just at the time when the fluorescein intensity became indistinguishable compared with that of the retinal arteries. Angiography was recorded with the built-in video equipment for the first 40 s, and then via serially shot photographs. Most of the data were obtained from the video recordings, and if the dye had not reached the disc within 40 s, the RC times were judged by the subsequently acquired serial photographs; in such case, we determined the RC time just before all the veins became indistinguishable in order not to overestimate RC time. When the RC time is different in affected and unaffected area, the shortest RC time was adopted. The RC time/AR time (RC/AR) ratio was calculated by dividing the RC time by the AR time.

### Statistical analyses

All results are expressed as the mean ± standard error. Commercially available software (SPSS version 26.0, IBM Corp., Armonk, NY, USA) was used for statistical analyses. Pearson’s Chi-square test and the Mann–Whitney *U* test were used for the analyses. The associations between AR time and RC time were calculated with Pearson’s correlation coefficient. Differences were considered statistically significant at P < 0.05.

## Supplementary Information

Supplementary Information.

## Data Availability

The datasets used and/or analysed during the current study available from the corresponding author on reasonable request.
