# Supplementary material for: Retinal circulation time/arm-to-retina time ratio in the fluorescein angiography to evaluate retina-specific hemodynamics
Source: Sci Rep. 2022 Oct 19;12:17457. doi: 10.1038/s41598-022-21117-3 (PMC9581933; doi:10.1038/s41598-022-21117-3)
Supplement: Supplementary file 1 — Supplementary Information. [file 41598_2022_21117_MOESM1_ESM.pdf]

Retinal circulation time/arm-to-retina time ratio in the fluorescein angiography to evaluate retina-specific hemodynamics

Yasuaki Mushiga[1,2,4], Norihiro Nagai [1,2,3,4], Yoko Ozawa [1,2,3,4,*]

[1]Department of Ophthalmology, St. Luke's International Hospital, 9-1 Akashi-cho, Chuo-ku, Tokyo 104-8560, Japan

[2]St. Luke's International University, 9-1 Akashi-cho, Chuo-ku, Tokyo 104-8560, Japan

[3]Laboratory of Retinal Cell Biology, Department of Ophthalmology, Keio University School of Medicine, 35 Shinanomachi, Shinjuku-ku, Tokyo 160-8582, Japan

[4]Department of Ophthalmology, Keio University School of Medicine, 35 Shinanomachi, Shinjuku-ku, Tokyo 160-8582, Japan

***Corresponding author:**
Yoko Ozawa, M.D., Ph.D.
Professor and Director
Department of Ophthalmology,
St. Luke's International University and Hospital
Lab Chief & Associate Professor
Laboratory of Retinal Cell Biology,
Department of Ophthalmology,
Keio University School of Medicine
E-mail: ozawa@a5.keio.jp, ozaway@luke.ac.jp
ORCID: 0000-0003-4797-5705

Supplementary Table 1. Characteristics in each disease

	DR	BRVO	CRVO	AMD	CSC	mCNV
Number of patients	37	22	4	37	18	10
Age (years old)	58.1 ± 11.2 (40 to 84)	68.8 ± 16.4 (48 to 89)	71.8 ± 9.9 (59 to 80)	72.5 ± 12.6 (42 to 93)	53.5 ± 14.1 (37 to 90)	62.5 ± 16.7 (49 to 92)
Sex (men [%])	28 (76)	14 (64)	1 (25)	26 (70)	12 (67)	6 (60)
BCVA (LogMAR)	0.19 ± 0.40 (-0.08 to 1.7)	0.19 ± 0.25 (-0.08 to 1.0)	0.16 ± 0.14 (-0.08 to 0.2)	0.24 ± 0.43 (-0.08 to 2.0)	0.15 ± 0.12 (-0.08 to 0.3)	0.33 ± 0.63 (-0.08 to 2.0)
Intraocular pressure (mmHg)	14.8 ± 3.5 (9.0 to 25.0)	12.5 ± 2.8 (9.0 to 19.0)	12.5 ± 1.7 (11.0 to 14.0)	13.5 ± 2.7 (9.0 to 19.0)	14.8 ± 3.7 (10.0 to 21.0)	14.6 ± 2.1 (12.0 to 18.0)
AR time (second)	16.1 ± 5.0 (8.0 to 25.6)	16.4 ± 4.0 (11.0 to 25.6)	15.8 ± 4.6 (12.2 to 22.2)	16.7 ± 3.8 (9.1 to 24.3)	15.2 ± 3.2 (10.9 to 22.7)	16.7 ± 3.3 (10.3 to 21.6)
RC time (second)	12.1 ± 4.1 (5.9 to 28.8)	10.8 ± 3.2 (6.9 to 18.0)	13.1 ± 4.1 (8.2 to 17.8)	10.2 ± 2.5 (7.2 to 18.4)	9.9 ± 2.6 (5.9 to 15.3)	9.5 ± 2.1 (6.8 to 12.2)
RC/AR ratio	0.79 ± 0.26 (0.28 to 1.43)	0.68 ± 0.20 (0.37 to 1.10)	0.83 ± 0.15 (0.64 to 0.98)	0.64 ± 0.19 (0.32 to 1.25)	0.66 ± 0.17 (0.44 to 1.03)	0.58 ± 0.14 (0.35 to 0.80)

Data are shown in mean ± standard error (range). AR time, arm-to-retina time; RC time, retinal circulation time; RC/AR ratio, RC time / AR time ratio. DR, diabetic retinopathy; BRVO, branch retinal vein occlusion; CRVO, central retinal vein occlusion; AMD, age-related macular degeneration; CSC, central serous chorioretinopathy; mCNV, myopic choroidal neovascularization.

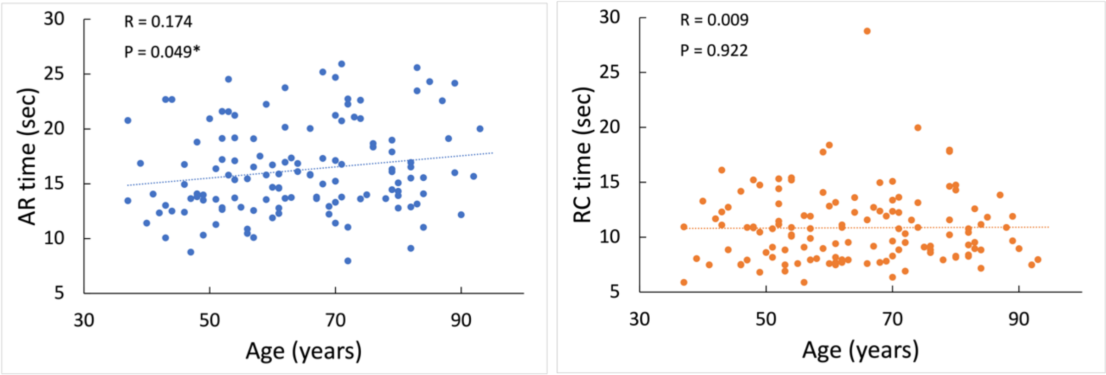

Supplementary Figure 1. Correlation between arm-to-retina (AR) time and age, and retinal circulation time and age in individual patients. A positive correlation was observed between AR time and age. * P <0.05.

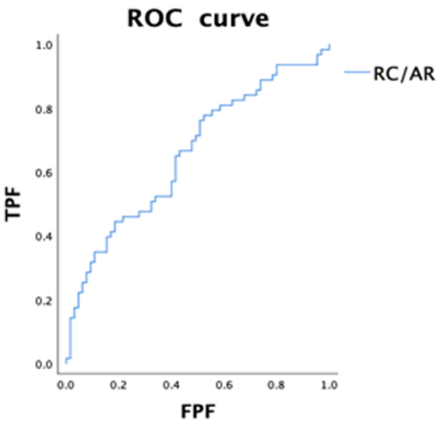

Supplementary Figure 2. Receiver Operating Characteristic (ROC) analysis of Retinal circulation (RC) time/ Arm-to-retina (AR) time ratio. TPF, true positive fraction; FPF, false positive fraction.
